# A Comprehensive Study on Non-Proprietary Ultra-High-Performance Concrete Containing Supplementary Cementitious Materials

**DOI:** 10.3390/ma16072622

**Published:** 2023-03-25

**Authors:** Seyedsaleh Mousavinezhad, Gregory J. Gonzales, William K. Toledo, Judit M. Garcia, Craig M. Newtson, Srinivas Allena

**Affiliations:** 1 Department of Civil Engineering, New Mexico State University, Las Cruces, NM 88003, USA; 2 Department of Civil and Environmental Engineering, Cleveland State University, Cleveland, OH 44115, USA

**Keywords:** durability, fly ash, ground granulated blast-furnace slag, metakaolin, natural pozzolan, Ultra-high performance concrete

## Abstract

Ultra-high performance concrete (UHPC) is a novel cement-based material with exceptional mechanical and durability properties. Silica fume, the primary supplementary cementitious material (SCM) in UHPC, is expensive in North America, so it is often substituted with inexpensive class F fly ash. However, future availability of fly ash is uncertain as the energy industry moves toward renewable energy, which creates an urgent need to find cost-effective and environmentally friendly alternatives to fly ash. This study investigated replacing cement, fly ash, and silica fume in UHPC mixtures with ground granulated blast-furnace slag (GGBFS), metakaolin, and a natural pozzolan (pumicite). To identify acceptable UHPC mixtures (28-day compressive strength greater than 120 MPa), workability, compression, and flexural tests were conducted on all mixtures. Then, durability properties including shrinkage, frost resistance, and chloride ion permeability (rapid chloride permeability and surface resistivity tests) were evaluated for the acceptable UHPC mixtures. Results showed that 75, 100, and 40% of fly ash in the control mixture could be replaced with pumicite, metakaolin, and GGBFS, respectively, while still producing acceptable strengths. Flexural strengths were greater than 14.20 MPa for all mixtures. For durability, UHPC mixtures had shrinkage strains no greater than 406 μstrain, durability factors of at least 105, and “very low” susceptibility to chloride ion penetration, indicating that these SCMs are suitable candidates to completely replace fly ash and partially replace silica fume in non-proprietary UHPC.

## 1. Introduction

Ultra-high performance concrete (UHPC) is an emerging concrete material with exceptional mechanical and durability properties, such as resistance to frost damage and chloride ion penetration [1]. UHPC must have a minimum 28-day compressive strength of 120 MPa and a minimum tensile strength (at first crack) of 6.9 MPa to meet the combined requirements of ASTM C1856 [2], the Canadian Standards Association (CSA A23.1 [3]), and the Swiss Society of Engineers and Architects (SIA 2052 [4]). Proprietary UHPC mixtures developed with specific high-quality materials are often shipped long distances, even internationally, for use on construction projects. However, the sustainability of this practice is questionable due to high production costs (up to 20 to 30 times that of normal strength concrete [NSC]) driven by expensive aggregates and steel fibers, high contents of high-quality cementitious materials that are not economically viable in all locations, and the shipping costs. Aggregate cost is often elevated due to processing high-quality sands (clean quartz or similar) to achieve an optimal gradation with a narrow range of particle sizes. Non-proprietary UHPC offers potential sustainability improvements by using local aggregates that may be of marginal quality with natural particle size distributions. Non-proprietary UHPC may also utilize inexpensive supplementary cementitious materials (SCMs) that are locally available but have lower quality than SCMs commonly used in proprietary UHPC, such as silica fume.

Silica fume is a key SCM in proprietary UHPC that improves density, mechanical properties, and durability properties because of its reactivity and its small particle size [5,6]. In many locations, silica fume has been partially replaced with class F fly ash because silica fume can be expensive in locations where it is not produced [7,8]. Unfortunately for the concrete industry, class F fly ash availability is diminishing because coal-fired power plants are being decommissioned [9,10]. Consequently, there is a need to identify replacements for silica fume that can reduce cost of locally produced UHPC.

Although a few studies have been conducted on non-proprietary UHPC mixtures [11,12,13], variability in potential constituent materials and their properties in different regions demands a larger body of research to further the development of these sustainable mixtures. Previous non-proprietary UHPC studies have typically used just one alternative SCM to replace the primary SCMs (silica fume and fly ash) in UHPC mixtures. Additionally, most have used either ultra-fine aggregates or an optimized gradation, both of which increase the cost of UHPC production. The narrow focus of individual research papers leaves a need for comprehensive studies to comparatively evaluate the effects of a broad range of SCMs on rheological, mechanical, and durability properties of non-proprietary UHPC mixtures, especially mixtures containing locally available aggregates with larger aggregate top size and with less processing to control gradation.

This research investigated the possibility of replacing cement, fly ash, and silica fume as primary constituents in non-proprietary UHPC due to the high costs and environmental impacts of silica fume and fly ash, respectively, in North America [7,8,9,10]. To replace the original cementitious materials, three SCMs that are considered to be more sustainable were used. The alternative SCMs included a natural pozzolan (pumicite), metakaolin, and GGBFS. These SCMs were selected because they are the most readily available and among the most sustainable SCMs in the USA. Specifically, pumicite is locally available in New Mexico, USA, where the research has been conducted. Although metakaolin and GGBFS are not locally produced in New Mexico, USA, they are available regionally.

To conduct the research, the effects of replacing cement, fly ash, and silica fume with pumicite, metakaolin, and GGBFS on workability, compressive strength, and flexural strength of 16 mixtures were assessed to identify mixtures that qualified as UHPC (compressive strength greater than 120 MPa and tensile strength at first crack greater than 6.9 MPa). After identifying acceptable UHPC mixtures, durability characteristics including shrinkage, frost resistance, rapid chloride permeability, and surface resistivity were investigated. It should be noted that a locally available, naturally occurring sand (4.75 mm top size) that is commonly used for concrete production in New Mexico, USA, was used without additional processing to control gradation.

## 2. Literature Review

This section reviews studies conducted on UHPC mixtures and the effects of SCMs, including natural pozzolans, metakaolin, and GGBFS on mechanical and durability properties of UHPC mixtures.

### 2.1. UHPC Sustainability

Studies have shown that the superior durability of UHPC has the potential to extend the service life of structures to more than two hundred years, which is two or three times greater than the service lives of the structures made with NSC [14,15]. Additionally, the high strength of UHPC (greater than 120 MPa) can facilitate significant reductions in the size of some concrete elements. According to Habert et al. [16], the CO_2_ emission of UHPC (using more than 1400 kg/m^3^ Portland cement) initially appears to be five to seven times greater than that of NSC when comparing the same amount of material. However, the environmental impact of UHPC can be less than 72% of the environmental impact from the NSC by considering the reduced concrete consumption and the improved service life.

### 2.2. Natural Pozzolan

A natural pozzolan is a raw or calcined natural material with pozzolanic properties that can lower both costs and carbon dioxide emissions for concrete. Pozzolanic materials are siliceous or siliceous and aluminous materials that can produce cementitious properties when they react with calcium hydroxide (Ca(OH)_2_) in the presence of water [17]. Studies on different types of concrete such as NSC and self-compacting concrete indicate that natural pozzolans can act as both filler and pozzolanic material in concrete and are capable of increasing rate of hydration, reducing heat of hydration, and improving durability properties, such as resistance to sulfate attack and alkali–silica reaction [18,19,20].

In UHPC mixtures, studies have shown that partially replacing cement (up to 30%) and silica fume (up to 50%) with a natural pozzolan resulted in UHPC specimens with very low (or negligible) chloride ion penetration and drying shrinkage of less than 500 μstrain, whereas workability and mechanical properties of these UHPC mixtures did not substantially change [21,22]. However, the literature reporting the effects of natural pozzolans in non-proprietary UHPC is severely lacking and each of these studies either used aggregates with a maximum size less than 1.2 mm, optimized gradation, or both [21,22,23]. Using ultra-fine aggregates or an optimal gradation increases UHPC production cost which negatively impacts sustainability. Another area where the literature is extremely lacking is in regards to replacing fly ash with natural pozzolan in non-proprietary UHPC mixtures.

### 2.3. Metakaolin

Metakaolin is an inexpensive SCM produced by calcination of kaolin. Since metakaolin is manufactured, its production can be tightly controlled to produce a consistent, highly reactive SCM. There are several studies on different types of concrete (not UHPC) containing metakaolin that highlight the positive effects of metakaolin on the mechanical and durability properties as well as sustainability of the concrete [24,25]. Additionally, the body of literature for UHPC mixtures containing metakaolin appears to be more extensive than for natural pozzolan. However, many of the papers have reported on mixtures that would not qualify as UHPC according to any widely used specification, such as ASTM C1856 [2], CSA A23.1 [3], or SIA 2052 [4]. Of the studies that produced acceptable UHPC, each used either aggregates with a top size of less than 1.25 mm, optimized gradation, or both [26,27,28,29]. As previously stated, using ultra-fine aggregates or an optimized gradation increases the cost of UHPC production. Additionally, there is little literature showing the effects of fly ash replacement with metakaolin in non-proprietary UHPC mixtures.

It has been found that replacing all of the silica fume (20% by mass of cementitious materials) with metakaolin reduced UHPC compressive strength by only 6.7% [27]. It should be noted that silica fume was the only SCM used in that study. In another study, inclusion of metakaolin in UHPC mixtures to replace silica fume resulted in acceptable durability properties [28]. It has also been shown that chloride permeability, time of set, workability, and shrinkage decreased with increasing metakaolin content in UHPC [18,21,26,29]. Using metakaolin in UHPC was found to reduce diffusion due to its high specific surface area and extremely fine particles [30]. In yet another study, replacing silica fume with a blend of metakaolin and fly ash led to better workability, greater 28-day compressive strength, and lower drying shrinkage [31].

### 2.4. Ground Granulated Blast-Furnace Slag

GGBFS is a highly cementitious by-product of iron extraction in a blast-furnace that appears to be a suitable alternative for cement, fly ash, and silica fume in UHPC. Although there are many studies on different types of concrete, but not UHPC, reporting the positive effects of GGBFS on early age strength, economic and environmental benefits, and service life [32,33,34,35], there are few studies on UHPC mixtures containing GGBFS, which is generally an inexpensive waste material. More importantly, previous studies have again used either an aggregate top size of less than 1.0 mm, optimal gradation, or both. As with natural pozzolan and metakaolin studies, there is little literature showing the effects of fly ash replacement with GGBFS in non-proprietary UHPC mixtures.

Previous UHPC studies aimed at minimizing silica fume content have not evaluated a broad range of rheological, mechanical, and durability properties. For instance, Ghafari et al. [36] focused only on shrinkage and reported that replacing 100% of the silica fume (by volume) with GGBFS in UHPC can decrease autogenous shrinkage without significantly changing compressive strength. There are studies that have evaluated a broad range of rheological, mechanical, and durability properties; however, those works maintained silica fume contents greater than 15% (by mass of cementitious materials) or only replaced Portland cement in their UHPC mixtures [37,38,39,40]. For instance, researchers showed that replacing 50% of the cement with GGBFS can produce 28-day compressive strengths comparable to the control mixtures (greater than 150 MPa) while increasing flowability and drying shrinkage in the first 24 h. Additionally, replacing cement with GGBFS was found to decrease short-term autogenous shrinkage while increase long-term autogenous shrinkage [37,38,39,40,41].

## 3. Research Significance

Silica fume and fly ash are two common sustainable SCMs in UHPC. Silica fume is expensive in the North America, and future availability of Class F fly ash is uncertain because of changes in the energy industry [7,8,9,10]. Although several studies have been conducted on non-proprietary UHPC mixtures, none of the journal research papers evaluated as many mechanical and especially durability properties as were considered in the present study. More importantly, some of the properties assessed in this study have not been reported at all in previous archival non-proprietary UHPC research papers.

There is a need for studies, like this one, that investigate a broad range of SCMs because previous non-proprietary UHPC studies have typically used just one alternative SCM to replace Portland cement or the primary SCMs (usually silica fume and occasionally fly ash) in non-proprietary UHPC mixtures. This paper also fills a need in the non-proprietary UHPC literature because most previous works have used either ultra-fine aggregates or an optimized gradation, both of which increase the cost of UHPC production. In this work, a natural gradation (using aggregates with larger aggregate top size compared to previous studies) was used because it produces a less expensive non-proprietary UHPC.

## 4. Materials and Methods

This section describes the materials used in this research and the methods used for mixing, assessment of workability, specimen preparation, compression and flexural testing, and durability testing that included assessment of shrinkage, frost resistance, rapid chloride permeability, and surface resistivity.

### 4.1. Materials

The sand used in this research had a maximum size of 4.75 mm, bulk specific gravity of 2.51, fineness modulus of 2.81, and absorption of 1.6% and was obtained locally in Las Cruces, New Mexico, USA. For cementitious materials, a Type I/II low-alkali Portland cement produced by GCC, a class F fly ash produced at the San Juan power plant in northern New Mexico, USA, a commercially available silica fume produced by BASF Construction Chemicals, Cambridge, USA (MasterLife SF 100), a natural pozzolan (pumicite) mined near Espanola, New Mexico, USA, a metakaolin product (GMK-S5) manufactured by Grace in Aiken, South Carolina, USA, and a GGBFS obtained from St. Marys Cement in Detroit, Michigan, USA were used in this research. Table 1 presents chemical and physical properties for the six cementitious materials used in this research. The steel fibers used in this study were NYCON-SF type 1 with 13 mm length, 0.2 mm diameter, and tensile strength of 1900 MPa. To achieve acceptable workability, a polycarboxylate-based high-range water-reducing admixture (HRWRA) produced by BASF Construction Chemicals, Cambridge, USA (MasterGlenium 3030 NS) was used.

### 4.2. Mixtures and Mixing Procedures

Table 2 provides all sixteen mixture proportions produced in this study. A UHPC mixture (F10/S10) developed in previous work [42] with 20% total SCM (10% silica fume and 10% fly ash, by mass of cementitious materials), water to cementitious materials (w/cm) ratio of 0.14, and 1.5% steel fibers (by total volume) was selected as the control mixture.

Because one of the main goals of this study was to replace fly ash and silica fume in UHPC with more sustainable SCMs, the study began with replacing 50 to 100% of the fly ash with a natural pozzolan and metakaolin. A limit of 40% fly ash replacement with GGBFS was selected because replacing more than 40% of the fly ash with GGBFS has been shown to produce less durable and workable mixtures with a 28-day compressive strength of less than 120 MPa [43,44]. Additionally, some ternary mixtures were produced to identify the effects of using a blend of various SCMs to replace fly ash. After replacing all of the fly ash from the control mixture, five mixtures were used to study the effects of replacing 10, 25, and 50% of the silica fume.

To interpret the mixture names, the letters indicate the SCM type in the mixture (F for fly ash, G for GGBFS, M for metakaolin, N for natural pozzolan, and S for silica fume), and the number after each letter indicates the percent of the SCM in the mixture. For instance, G2/M6/N2/S10 indicates a mixture with 2% GGBFS, 6% metakaolin, 2% natural pozzolan, and 10% silica fume as percentages of the total cementitious materials.

**Figure 1:**
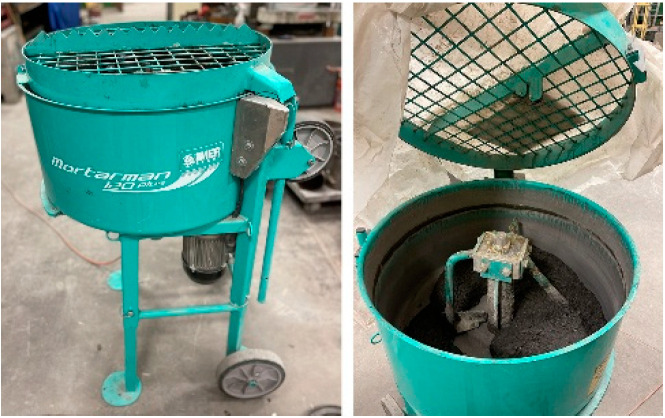
UHPC mixer.

This study utilized a pan mixer (Imer Mix 120 Plus) with a vertical shaft and three paddles in different locations (near, intermediate, and far locations relative to the shaft) and a mixing speed of 38 rpm. A photograph of the mixer is shown in Figure 1. The mixing sequence began with sand and cementitious materials for each mixture being placed in a pan mixer prior to starting the mixer. After starting the mixer, water and HRWRA were added with the mixer running. Then, the top of the mixer was covered to prevent evaporation. After 15 min of mixing, or when the mixture appeared to be uniformly mixed, whichever was longer, steel fibers were added, and the mixer was run for five additional mins before discharging the batch. For each mixture, a 0.057 m^3^ batch was used to produce six 100 mm cube specimens, five 102 by 203 mm cylindrical specimens, and eight 76 × 102 × 406 mm prismatic specimens, three of which had gauge studs (contact points) embedded in each end. The specimens were removed from the molds 24 h after casting and moist cured at 98% relative humidity (RH) and a temperature of 23 ± 2 °C for up to 56 days.

### 4.3. Fresh Properties

Fresh mixture properties were evaluated immediately after mixing by conducting slump and slump flow tests according to ASTM C143 [45] and ASTM C1611 procedure A [46], respectively. Target slump values were between 100 mm and 200 mm and target slump flow values were between 200 mm and 400 mm.

### 4.4. Mechanical Properties

To identify acceptable UHPC mixtures, the compressive and flexural strengths of all mixtures that had slump and slump flow values within the target ranges were assessed using a 1780 kN capacity universal testing machine.

#### 4.4.1. Compression Testing

According to ASTM C1856 [2], CSA A23.1 [3], and SIA 2052 [4], mixtures with a 28-day compressive strength greater than 120 MPa can be classified as UHPC. To assess compressive strength, three 100 mm cube specimens were tested for 28- and 56-day compressive strength according to BS1881 [47]. BS1881 [47] requires cubes to be tested at a load rate of 0.2 MPa/s to 0.4 MPa/s. However, the load rate in this study was increased to 1.0 MPa/s as stated by ASTM C1856 [2]. It should be noted that the reason for selecting cube specimens (instead of cylindrical specimens) for compression tests is that cubes do not require end preparation (grinding).

#### 4.4.2. Flexural Testing

**Figure 2:**
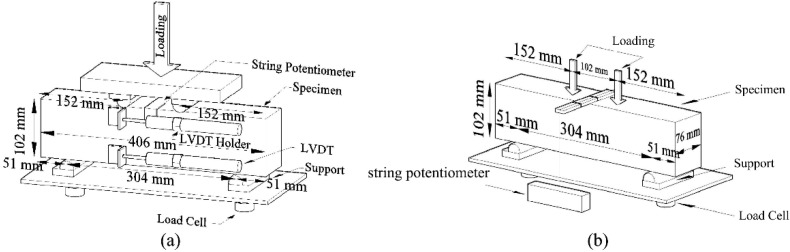
Schematic of flexural test: (**a**) front view and (**b**) back view.

According to CSA A23.1 [3] and SIA 2052 [4], UHPC must have a minimum 28-day tensile strength (at first crack) of 6.9 MPa. To evaluate flexural strength properties, two 76 × 102 × 406 mm prisms from each UHPC mixture were tested according to the flexural test described in ASTM C1609 [48]. Two load cells, a string potentiometer, and two linear variable differential transformers (LVDTs) were used to measure the load, mid-span deflection, and strains near the top and bottom of the specimens during flexural testing, as illustrated in Figure 2. Modulus of rupture (MOR), ultimate (peak) strength, residual strengths (including *f*_600_ and *f*_150_), and toughness at 28 days were computed as described in ASTM C1609 [48].

### 4.5. Durability Properties

After identifying acceptable UHPC mixtures, cement, fly ash, or silica fume were replaced with alternative SCMs and durability properties that included shrinkage, frost resistance, and chloride ion permeability were evaluated.

#### 4.5.1. Shrinkage Testing

Three prisms with dimensions of 76 × 102 × 406 mm from each UHPC mixture were used for shrinkage testing. Specimens were cured for 28 days in a moist room with RH of 96% and temperature of 23 ± 2 °C and then stored in ambient conditions (RH of 30 ± 4% and temperature of 20 ± 2 °C) for an additional 28 days. The RH of 30% was less than the 50% recommended by ASTM C157 [49], and therefore, provided conservative shrinkage results. Shrinkage was measured by monitoring length changes for 56 days using a length comparator.

#### 4.5.2. Freezing and Thawing Testing

**Figure 3:**
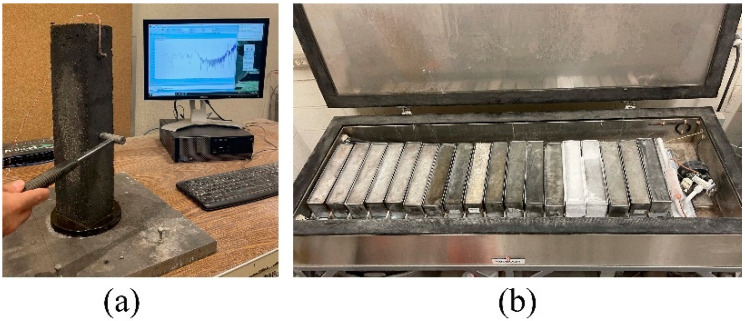
Freezing and thawing test: (**a**) fundamental frequency test and (**b**) freeze-thaw chamber.

To assess frost resistance of UHPC mixtures according to ASTM C666 [50], mass and fundamental transverse frequency were measured (Figure 3a) for three 76 × 102 × 406 mm prisms from each UHPC mixture exposed to 300 cycles of freezing and thawing (Figure 3b) at intervals no greater than 36 cycles. Fundamental frequency was determined using the impact resonance method (ASTM C215 [51]) by striking a specimen with an instrumented hammer and recording the acceleration response history using a lightweight accelerometer attached to the specimen (Figure 3a). Fundamental frequency, resonant frequency for first mode vibration, was determined by identifying the frequency at which the maximum acceleration response occurred from a plot of amplitude versus frequency.

Fundamental transverse frequency and mass were used to calculate dynamic elastic modulus (*E_D_*) according to ASTM C215 [51]:

(1) $$E_{D}=C\cdot M\cdot n^{2}$$

where *C* is a constant (1114 m^−1^) determined from the geometry of the specimen and an assumed Poisson’s ratio (0.167), *M* is mass (kg), and n is fundamental transverse frequency (Hz). Then, relative dynamic modulus (RDM) was computed as:

(2) $$RDM=\frac{E_{n}}{E_{0}}\times 100$$

where *E_n_* and *E*_0_ are dynamic elastic modulus (Pa) values after n and 0 cycles, respectively (ASTM C666 [50]). Durability factor (DF), *RDM* after 300 cycles of freezing and thawing, was used to compare the frost resistance of UHPC mixtures.

#### 4.5.3. Rapid Chloride Permeability Testing

**Figure 4:**
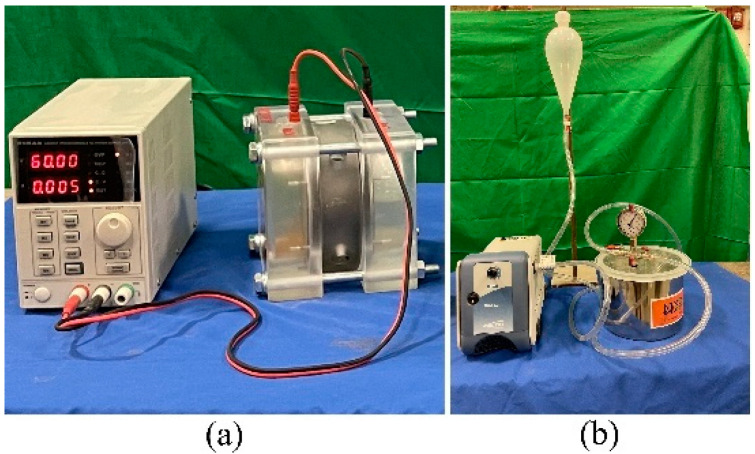
RCPT: (**a**) power supply and test cell and (**b**) vacuum pump and desiccator.

The rapid chloride permeability test (RCPT), ASTM C1202 [52], is commonly used to evaluate the chloride ion permeability of concrete. Two 51-mm slices cut from two 102 by 203 mm cylinder specimens were prepared for testing using a vacuum desiccator in accordance with ASTM C1202 [52]. After preparation, specimens were placed in a testing cell with one side exposed to a 3.0% sodium chloride solution and the other side exposed to a 0.3 N sodium hydroxide solution. This test was performed using a 60 V DC power supply to pass a current through the specimen that was measured every 30 min for six hours. Current measurements were used to calculate total charge passed (coulombs) according to ASTM C1202 [52]. RCPTs were conducted as shown in Figure 4 at an age of 56 days in accordance with ASTM C1202 [52].

#### 4.5.4. Surface Resistivity Testing

**Figure 5:**
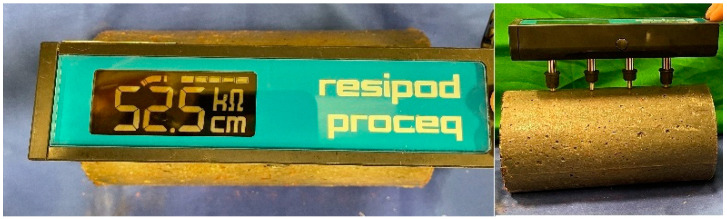
Surface resistivity test.

**Figure 6:**
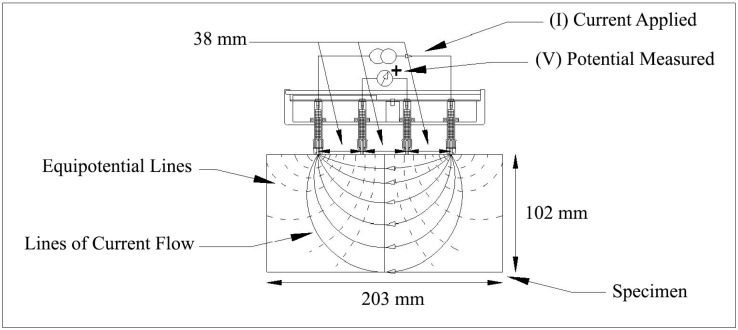
Four-point Wenner probe array test setup.

The surface resistivity test is another standard test that provides a rapid indication of chloride ion permeability for concrete (AASHTO T 358 [53]). Three 102 by 203 mm cylinder specimens were tested for surface resistivity using a 4-Pin Wenner probe array (Resipod Proceq) according to AASHTO T 358 [53], as shown in Figure 5. In this test, conducted at an age of 56 days, an alternating current potential difference was applied by the outer pins (current electrodes) of a four-pin Wenner probe array and the resultant potential difference between the two inner pins (potential electrodes) was measured (Figure 6). The current used, the resulting potential, and the affected specimen area were used to determine surface resistivity.

## 5. Results and Discussion

This section presents the results from mechanical and durability testing performed in this research to assess workability, compressive strength, flexural strength, shrinkage, frost resistance, and chloride ion permeability.

### 5.1. Workability

Table 2 presents the slump and slump flow results for each mixture that had an acceptable slump (between 100 mm and 200 mm) and an acceptable slump flow (between 200 mm and 400 mm).

Results indicate that substituting pumicite, metakaolin, and GGBFS for fly ash increased HRWRA demand (decreased workability). This can be attributed to pumicite, metakaolin, and GGBFS being more angular than fly ash, which has spherical particles that create a ball-bearing effect in the fluid state [44,54,55]. Pumicite and metakaolin have greater specific surface areas than fly ash (Table 1) that lead to greater water absorption rates [56,57]. Replacing all of the fly ash in the control mixture with natural pozzolan (mixture N10/S10), all of the fly ash in the control mixture with metakaolin (mixture M10/S10), and 40% of the fly ash in the control mixture with GGBFS (mixture G4/F6/S10) required 10.0, 20.0, and 10.0% more HRWRA, respectively, to achieve acceptable workability. From these results, replacing all of the fly ash with pumicite increased HRWRA demand as much as replacing 40% of the fly ash with GGBFS to obtain acceptable slump and slump flow values, indicating that GGBFS decreased workability more than pumicite. Additionally, mixtures with metakaolin needed additional HRWRA to achieve the target slump and slump flow values compared with mixtures with pumicite. Since UHPC workability generally depends on particle size (specific surface area) and shape of ingredients, ultra-fine metakaolin with smaller particles (greater specific surface area) than pumicite has a greater water absorption rate that leads to an increase in HRWRA demand.

In the present research, comparing mixtures M10/N2.5/S7.5, M12.5/S7.5, and M15/S5 with mixture M10/S10 shows that replacing silica fume with either pumicite or metakaolin reduced demand for HRWRA. This can be attributed to the silica fume particles being smaller and requiring additional water due to their greater surface area than pumicite and metakaolin particles [56,57].

### 5.2. Compression Tests

**Figure 7:**
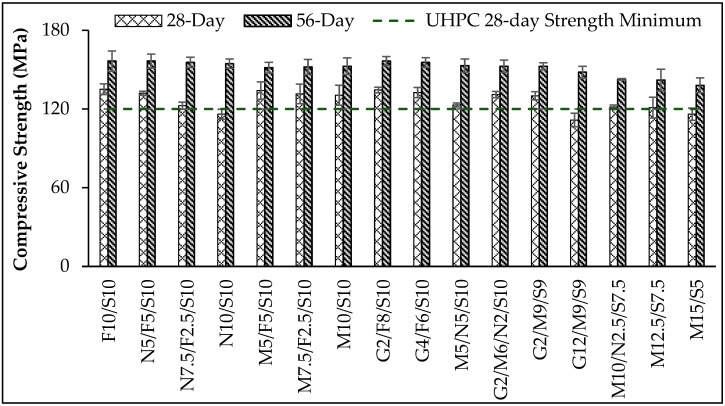
Compressive strength results.

Average 28- and 56-day compressive strengths for three 100 mm cubes from each mixture are presented in Figure 7 and Table 3. Mixtures N10/S10, G12/M9/S9, and M15/S5 did not produce compressive strengths greater than 120 MPa at 28 days, which is a requirement for UHPC based on ASTM C1856 [2], CSA A23.1 [3], and SIA 2052 [4]. However, the 56-day strengths for these mixtures were greater than 138.0 MPa, which is suitable for many UHPC applications, demonstrating that more flexible requirements for defining UHPC could exploit long-term benefits of the pumicite, metakaolin, or GGBFS.

The control mixture (F10/S10) had the greatest 28-day compressive strength among all mixtures, meaning that replacing fly ash with natural pozzolan, metakaolin, or GGBFS decreased 28-day compressive strength. Replacing all of the fly ash in the control mixture with natural pozzolan (N10/S10) or metakaolin (M10/S10) reduced compressive strength by 13.9 and 3.0%, respectively. This observation was expected because fly ash reacts more quickly than the alternative SCMs used in this study [26,58], which is the primary reason why fly ash has been commonly used as a SCM in non-proprietary UHPC mixtures. It should be stated again that the diminishing availability of fly ash in the USA is the only reason to replace fly ash with other SCMs in this study.

Substantial reduction in 28-day compressive strength of pumicite mixtures (up to 13.9%) indicated that the natural pozzolan reacts slowly compared with fly ash, for at least 28 days, which is consistent with previous research [26,58]. However, at 28 days, the metakaolin and GGBFS reactions had nearly caught up with the reaction of fly ash in the control mixture (less than 3.0% reduction in 28-day compressive strength). Replacing all of the fly ash in the control mixture with 50% natural pozzolan and 50% metakaolin (M5/N5/S10) reduced 28-day compressive strength by 8.6%, showing that the reaction of the pumicite and metakaolin was not rapid enough to catch up with the reaction of fly ash during the first 28 days.

It is generally expected that replacing Portland cement in UHPC with metakaolin increases compressive and flexural strengths, whereas replacing silica fume with metakaolin slightly reduces compressive and flexural strengths [27,59]. Results from mixtures M10/N2.5/S7.5, M12.5/S7.5, and M15/S5 indicate that replacing 25% of the silica fume with pumicite, 25% of the silica fume with metakaolin, and 50% of the silica fume with metakaolin reduced 28-day compressive strengths by 11.9, 10.2, and 13.9%, respectively, compared with mixture M10/S10. As stated previously, replacing all of the fly ash in the control mixture with the natural pozzolan (N10/S10) or metakaolin (M10/S10) reduced 28-day compressive strength by 13.9 and 3.0%, respectively. Replacing 25% of the silica fume with pumicite or metakaolin decreased compressive strength more than (or just slightly less than) when 100% of the fly ash was replaced with pumicite or metakaolin indicates that silica fume is a more reactive SCM than fly ash in terms of UHPC strength development during the first 28 days.

Results from 56-day compressive strengths show that reductions in strengths caused by replacing fly ash (but not silica fume) with alternative SCMs were of less magnitude than reductions at 28 days. The greatest reduction (3.3%) occurred when 50% of the fly ash in the control mixture was replaced with metakaolin (M5/F5/S10). This small reduction shows that the natural pozzolan, metakaolin, and GGBFS produced comparable 56-day compressive strengths to F10/S10 (control mixture). As a reminder, metakaolin and GGBFS mixtures also had comparable compressive strengths to the control mixture at 28 days. However, the fact that pumicite mixtures had comparable 56-day compressive strengths to the control mixture indicate that the natural pozzolan reaction had finally progressed to a point of equivalency with the fly ash reaction. This occurred because the natural pozzolan was more reactive than fly ash after 28 days, which is consistent with observations by other researchers [54,58]. Due to the diminishing availability of fly ash, lower compressive strengths at 28 days for mixtures containing natural pozzolan may need to be tolerated to capture the economic and durability benefits of the natural pozzolan.

Although compression tests showed that replacing silica fume with natural pozzolan or metakaolin reduced compressive strengths at 56 days, mixtures with up to 50% replacement of silica fume had 56-day compressive strengths greater than 138.0 MPa, indicating that longer-term benefits of these mixtures are probably worth evaluating in future research.

Comparing mixtures G2/M9/S9 and G12/M9/S9 shows that replacing 10% of the cement in mixture G2/M9/S9 with GGBFS (after replacing all of the fly ash and some of the silica fume) decreased compressive strengths at 28 and 56 days by 14.3 and 3.0%, respectively. This shows that GGBFS is less reactive than cement in the first 28 days and that GGBFS needs more time to produce compressive strengths comparable to the cement that it replaced.

### 5.3. Flexural Tests

Table 4 presents the average flexural properties from each mixture. The results show that all mixtures had tensile strength at first peak (MOR) values greater than 6.9 MPa, so all mixtures can be classified as UHPC according to CSA A23.1 [3] and SIA 2052 [4]. Trends for MOR, peak (ultimate), and residual strength (*f*_600_ and *f*_150_) values with changing SCMs contents was consistent with the trends observed for 28-day compressive strengths. Replacing fly ash (but not silica fume) with any of the alternative SCMs used in this study produced either better or comparable flexural strength compared to the control mixture, with MOR, peak strength, and toughness values greater than 18.50 MPa, 19.30 MPa, and 65 J, respectively. This shows that the natural pozzolan, metakaolin, and GGBFS are promising substitutes for fly ash in terms of flexural strength. However, replacing cement or silica fume with alternative SCMs (after replacing all of the fly ash) significantly reduced flexural strength, although it remained greater than 14.20 MPa, which is acceptable for most UHPC applications.

Fibers contribute substantially to UHPC toughness (energy absorbed for a deflection equal to 0.667% of the span length) and because the volumetric content and properties of the steel fibers were the same for all mixtures, similar toughness results were expected for all mixtures. However, substantial loss in toughness occurred when more than 25% of the silica fume was replaced with alternative SCMs, showing the importance of silica fume for developing toughness.

### 5.4. Shrinkage Tests

**Figure 8:**
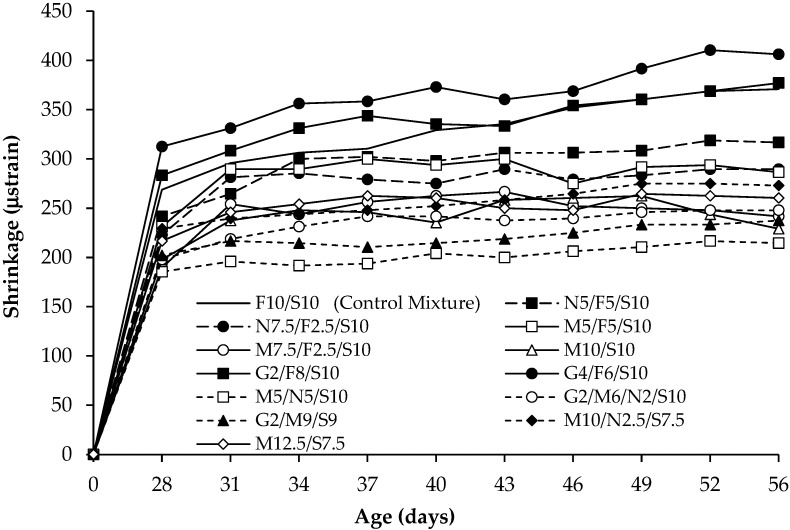
Shrinkage results.

Figure 8 shows average shrinkage results of three prisms from each UHPC mixture. After demolding, shrinkage was monitored for 56 days (28 days of moist curing followed by 28 days of air curing). The results indicate that the greatest shrinkage occurred when replacing 40% of the fly ash with GGBFS (G4/F6/S10) where 56-day shrinkage was 406 μstrain, showing that all of the UHPC mixtures had shrinkage values of less than the 500 μstrain that is acceptable to many transportation agencies. The fact that the UHPC mixtures had little susceptibility to shrinkage is consistent with previous research [22,58]. Because UHPC has high cementitious materials contents, it was expected that shrinkage would be greater than what was observed in this study. It appears that the steel fibers provide internal restraint and the low w/cm ratio caused incomplete hydration of cementitious materials which led to limited shrinkage.

Replacing 75% of the fly ash in the control mixture (F10/S10) with natural pozzolan (N7.5/F2.5/S10), all of the fly ash in F10/S10 with metakaolin (M10/S10), and all of the fly ash in F10/S10 with a combination of natural pozzolan and metakaolin (M5/N5/S10) reduced 56-day shrinkage by 21.8, 38.3, and 42.0%, respectively. Additionally, increasing natural pozzolan from 5 to 7.5% in mixtures N5/F5/S10 and N7.5/F2.5/S10 or increasing metakaolin from 5 to 10% in mixtures M5/F5/S10, M7.5/F2.5/S10, and M10/S10 reduced shrinkage. These trends were consistent with previous research [22,26,31] and can be attributed to pumicite and metakaolin particles being much smaller (greater surface area) than fly ash particles (Table 1) and causing greater refinement of the capillary pores that reduces drying shrinkage by obstructing evaporation of capillary water [22]. Another mechanism to reduce shrinkage is the slow reactivity of the pumicite that can delay shrinkage until the skeletal structure has formed and helps restrain shrinkage.

Replacing 40% of the fly ash in the control mixture with GGBFS (G4/F6/S10) increased shrinkage. Previous research also confirmed that using GGBFS in UHPC increases shrinkage [38,41]. This can be attributed to GGBFS being more angular and slightly larger than the spherical fly ash particles, which can lead to less refined capillary pores.

Comparing mixtures M10/N2.5/S7.5 and M12.5/S7.5 with mixture M10/S10 indicates that adding pumicite or more metakaolin to replace 25% of the silica fume (after replacing all of the fly ash in the control mixture with metakaolin to produce mixture M10/S10) increased shrinkage at 56 days by 19.2 and 13.5%, respectively, showing that replacing silica fume with pumicite or metakaolin increases shrinkage, which is consistent with arguments from other researchers that silica fume with smaller particles (greater surface area) than pumicite and metakaolin is better for limiting shrinkage by filling capillary pores and blocking pathways for evaporating capillary water [22].

### 5.5. Freezing and Thawing Tests

**Figure 9:**
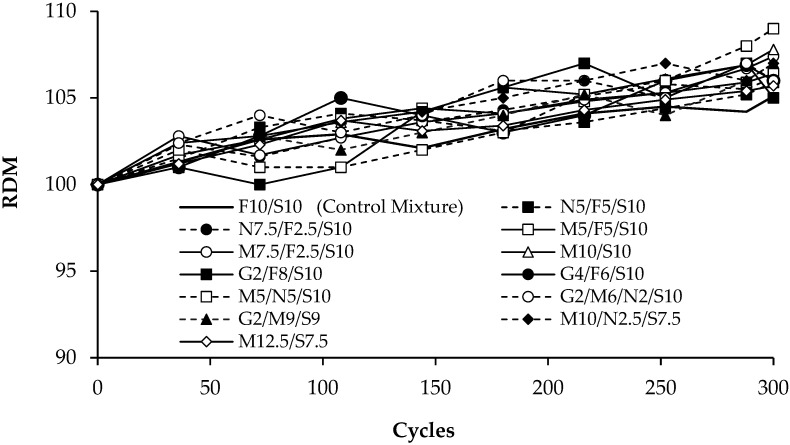
Freezing and thawing test results.

Results for freezing and thawing tests are presented in Figure 9. The results show that DF for all UHPC mixtures were greater than 105 and the greatest DF values (109) were produced by mixtures M10/S10 and M5/N5/S10. This shows that all of the UHPC mixtures were extremely resistant to degradation caused by freezing and thawing cycles, regardless of SCM type. Other researchers have also shown that UHPC has excellent resistance to freezing and thawing [60].

The results also show that RDM values increased slightly during the freezing and thawing cycles, which can be attributed to specimens containing un-hydrated cementitious materials that were able to react throughout the duration of the test, causing an increase in dynamic modulus [60,61]. However, RDM values for all UHPC mixtures were similar and replacing fly ash or silica fume in the control mixture with natural pozzolan, metakaolin, GGBFS, or a combination of these SCMs did not significantly change the RDM and DF values. This shows that pumicite, metakaolin, and GGBFS all appear to be suitable substitutes for fly ash and silica fume in terms of frost resistance.

### 5.6. Chloride Ion Permeability Tests

The chloride ion permeability of each UHPC mixture was evaluated with RCPTs and surface resistivity tests at 56 days according to ASTM C1202 [52] and AASHTO T 358 [53], respectively. It is recommended to moist cure specimens containing SCMs for at least 56 days prior to performing permeability tests (ASTM C1202 [52]) to allow slow-reacting SCMs to react more completely and provide a better indication of the long-term chloride ion permeability. Average 56-day RCPT and surface resistivity test results from each UHPC mixture are presented in Table 5. The results showed that total charge passed (RCPT results) were in the range of 119 and 165 coulombs and surface resistivity results ranged between 386 kΩ-mm and 603 kΩ-mm. These results show that the chloride ion penetrations for all of the UHPC mixtures were “very low” (ASTM C1202 [52]; AASHTO T 358 [53]), which is consistent with observations from other researchers [22,29,58].

Replacing fly ash with any of the alternative SCMs used in this study decreased chloride ion permeability (decreased total charge passed and increased surface resistivity). The greatest reduction in chloride ion permeability was produced by mixture M10/S10 when all of the fly ash in F10/S10 was replaced with metakaolin, with a 27.9% reduction in total charge passed and a 56.2% increase in surface resistivity. These results are consistent with observations of pumicite and metakaolin causing reduced chloride ion permeability by other researchers [22,58,62] that attribute the reduction to the SCMs forming additional secondary calcium silicate hydrate and fine pumicite and metakaolin particles filling pore space that leads to lower permeability.

After replacing all of the fly ash in F10/S10 with metakaolin (to produce mixture M10/S10), adding 25% more pumicite (M10/N2.5/S7.5) or metakaolin (M12.5/S7.5) to replace silica fume substantially increased permeability compared with mixture M10/S10, but the chloride ion penetration remained in the “very low” category. This increase in permeability, by replacing silica fume with pumicite or metakaolin, is most likely due to the pumicite and metakaolin particles being larger than silica fume particles (Table 1) and producing less refined capillary pores [22]. Overall, the results show that pumicite (natural pozzolan), metakaolin, and GGBFS appear to have the potential to partially or completely replace fly ash and silica fume in non-proprietary UHPC mixtures in terms of chloride ion permeability.

It should be noted that ASTM C1202 [52] and AASHTO T 358 [53] warn that specimens containing steel fibers or other embedded electrically conductive materials may produce erroneous RCPT and surface resistivity test results, with artificially high total charge passed and low surface resistivity values. However, the relatively low values in RCPT (less than 165 coulombs) and relatively large values in surface resistivity (greater than 386 kΩ-mm) obtained in this research as well as the consistency between RCPT and surface resistivity test results indicate that it is unlikely that a conductive path was created between the two ends of the specimens (in RCPTs) or Wenner probe pins (in surface resistivity tests). Other researchers have also reported that steel fibers in UHPC are generally dispersed and discontinuous and do not provide a direct path to complete an electric circuit [61].

### 5.7. RCPT and Surface Resistivity Test Comparison

RCPT and surface resistivity test results consistently indicated that replacing fly ash with natural pozzolan, metakaolin, or GGBFS reduced permeability, whereas replacing silica fume with pumicite or metakaolin increased permeability. This consistency between the RCPT and surface resistivity test results, in combination with the results being consistent with results from other researchers, show that both tests appear to be valid for assessing the chloride ion permeability of UHPC containing steel fibers [63]. However, the coefficients of variation for the surface resistivity test results (Table 5) were much greater than the maximum permissible value of 6.3% specified by AASHTO T 358 [53] for a single operator, whereas the coefficients of variation for the RCPT results were acceptable according to ASTM C1202 [52]. The high variation for the surface resistivity tests was most likely caused by the steel fibers; however, additional research would need to be performed to verify this.

## 6. Conclusions

Based on the results from this study, the following conclusions were drawn:

1. The targeted workability was achieved in no more than two attempts for all mixtures. A specific observation related to workability was that silica fume replacement with either metakaolin or pumicite reduced the required HRWRA dosage to achieve the target workability because the finer silica fume particles had greater surface area than the pumicite and metakaolin particles. Similar behavior was observed when metakaolin mixtures required more HRWRA dosage than pumicite mixtures, due to metakaolin having smaller particles than pumicite.
2. Natural pozzolan was substituted for up to 75% of the fly ash and metakaolin was used to replace up to 100% of the fly ash and 25% of the silica fume in the control mixture while producing 28-day compressive strengths greater than 120 MPa.
3. Mixtures with low 28-day compressive strength (less than 120 MPa) had 56-day compressive strengths that were greater than 138.0 MPa, indicating that lower 28-day compressive strengths may need to be tolerated to realize the economic and durability benefits of some alternative SCMs.
4. Replacing fly ash or silica fume in all mixtures with natural pozzolan, metakaolin, or GGBFS produced toughness values greater than 49 J and modulus of rupture values were at least 14.20 MPa, which is acceptable for most UHPC applications.
5. The UHPC mixtures had little susceptibility to shrinkage (none were greater than 406 μstrain) and chloride permeability (all mixtures had “very low” permeability) and also had excellent resistance to frost damage (all DF values were at least 105).
6. The results showed that pumicite (natural pozzolan), metakaolin, and GGBFS can replace fly ash without a substantial loss of strength and can even improve durability characteristics such as shrinkage, frost resistance, and chloride permeability.
7. Using pumicite or metakaolin to partially replace silica fume (after replacing all of the fly ash with metakaolin) improved durability properties compared with the control mixture but showed decreasing durability and strength properties with decreasing silica fume content.

## Figures and Tables

**Table 1:** Chemical and physical properties of cement and SCMs (% mass).

Chemical Compounds	Material					
	Cement Type I/II	Class F Fly Ash	Silica Fume	Pumicite	Metakaolin	GGBFS
CaO	63.9	8.99	0.3	0.40	0.87	38.44
SiO_2_	20.3	53.16	96.9	76.29	63.86	42.87
Al_2_O_3_	4.6	24.64	0.2	12.13	31.11	11.61
Fe_2_O_3_	3.4	4.22	0.2	1.74	1.06	0.92
MgO	1.91	1.25	0.2	0.07	0.18	4.29
Na_2_O	0.23	1.66	0.2	4.23	1.08	0.96
K_2_O	0.38	1.24	0.3	4.29	0.09	0.12
TiO_2_	-	-	-	0.10	-	-
MnO_2_	-	-	-	0.08	-	-
P_2_O_5_	-	-	-	0.02	-	-
SrO	-	-	-	0.01	-	-
BaO	-	-	-	0.01	-	-
SO_3_	2.86	0.25	0.1	0.00	0.05	0.17
Loss on Ignition	2.24	-	2.17	-	1.18	1.96
Physical Properties						
Specific Gravity	3.15	1.91	2.20	2.45	2.60	2.91
Spec. Surface Area (m^2^/kg)	335	734	26,810	17,348	22,320	542
Autoclave Expansion (%)	0.05	0.01	-	-	-	0.06

**Table 2:** Concrete mixture proportions and workability results.

Mixture Name	Cement (kg/m^3^)	Silica Fume (kg/m^3^)	Fly Ash (kg/m^3^)	Pumicite (kg/m^3^)	Metakaolin (kg/m^3^)	GGBFS (kg/m^3^)	Sand (kg/m^3^)	HRWRA (kg/m^3^)	Slump (mm)	Slump Flow (mm)
**F10/S10** **(Control Mixture)**	839	104	104	0	0	0	919	59.3	120	380
**N5/F5/S10**	839	104	52	52	0	0	925	62.3	115	370
**N7.5/F2.5/S10**	839	104	26	78	0	0	931	62.3	100	370
**N10/S10**	839	104	0	104	0	0	931	65.3	100	370
**M5/F5/S10**	839	104	52	0	52	0	913	68.2	130	380
**M7.5/F2.5/S10**	839	104	26	0	78	0	921	68.2	115	370
**M10/S10**	839	104	0	0	104	0	922	71.2	110	370
**G2/F8/S10**	839	104	83	0	0	21	920	62.3	120	380
**G4/F6/S10**	839	104	62	0	0	42	921	65.3	110	370
**M5/N5/S10**	839	104	0	52	52	0	926	68.2	115	370
**G2/M6/N2/S10**	839	104	0	21	62	21	923	71.2	110	360
**G2/M9/S9**	839	94	0	0	94	21	926	71.2	100	360
G12/M9/S9 *	755	94	0	0	94	105	928	68.2	125	380
**M10/N2.5/S7.5**	839	78	0	26	104	0	940	65.3	115	370
**M12.5/S7.5**	839	78	0	0	130	0	934	68.2	110	370
**M15/S5**	839	52	0	0	156	0	946	65.3	135	390

Note: Water and steel fiber contents of 147 and 119 kg/m^3^, respectively, were used for all mixtures. * In this mixture, an additional 10% of the cement was replaced with GGBFS.

**Table 3:** Compressive strength results (±standard deviation).

Mixture Name	28-Day (MPa)	56-Day (MPa)
**F10/S10 (Control Mixture)**	135.0 (±4.1)	156.5 (±7.7)
**N5/F5/S10**	132.0 (±1.6)	156.5 (±5.3)
**N7.5/F2.5/S10**	122.5 (±2.7)	155.5 (±3.9)
**N10/S10**	116.0 (±3.8)	154.5 (±3.6)
**M5/F5/S10**	134.0 (±6.6)	151.5 (±4.1)
**M7.5/F2.5/S10**	131.5 (±7.4)	152.0 (±5.8)
**M10/S10**	130.5 (±7.6)	152.5 (±6.4)
**G2/F8/S10**	134.5 (±2.1)	156.5 (±3.5)
**G4/F6/S10**	132.5 (±3.9)	155.5 (±3.6)
**M5/N5/S10**	123.0 (±1.5)	153.0 (±5.0)
**G2/M6/N2/S10**	131.0 (±2.3)	152.5 (±4.7)
**G2/M9/S9**	130.0 (±3.2)	152.5 (±2.7)
**G12/M9/S9**	111.5 (±5.2)	148.0 (±4.5)
**M10/N2.5/S7.5**	121.5 (±1.5)	142.5 (±0.7)
**M12.5/S7.5**	121.0 (±7.9)	142.0 (±8.3)
**M15/S5**	116.0 (±4.7)	138.0 (±5.7)

**Table 4:** Flexural strength results (±standard deviation).

Mixture Name	MOR (MPa)	Peak Strength (MPa)	Residual Strength (*f*_600_) (MPa)	Residual Strength (*f*_150_) (MPa)	Toughness (J)
**F10/S10 (Control Mixture)**	18.65 (±1.80)	19.55 (±1.50)	18.55 (±1.45)	10.85 (±0.10)	67 (±5)
**N5/F5/S10**	20.05 (±0.80)	21.25 (±1.00)	20.35 (±0.80)	11.20 (±0.85)	70 (±3)
**N7.5/F2.5/S10**	19.25 (±1.05)	20.80 (±1.20)	19.75 (±0.85)	11.05 (±0.35)	70 (±1)
**N10/S10**	18.75 (±1.30)	20.00 (±1.35)	18.70 (±0.85)	10.15 (±0.55)	67 (±2)
**M5/F5/S10**	19.50 (±0.45)	20.90 (±0.55)	20.05 (±0.60)	10.25 (±0.70)	68 (±1)
**M7.5/F2.5/S10**	19.20 (±0.50)	20.50 (±0.60)	19.60 (±0.10)	9.95 (±0.70)	67 (±1)
**M10/S10**	18.85 (±0.95)	20.40 (±0.85)	19.50 (±0.70)	6.70 (±0.40)	65 (±1)
**G2/F8/S10**	18.60 (±1.40)	19.50 (±0.75)	18.45 (±0.70)	10.70 (±0.20)	67 (±5)
**G4/F6/S10**	18.50 (±0.85)	19.30 (±1.00)	17.95 (±0.70)	10.25 (±0.30)	66 (±2)
**M5/N5/S10**	20.65 (±1.25)	22.25 (±1.50)	21.80 (±2.05)	11.70 (±0.70)	75 (±5)
**G2/M6/N2/S10**	19.50 (±0.70)	21.15 (±0.35)	20.25 (±0.15)	10.60 (±0.85)	72 (±2)
**G2/M9/S9**	19.40 (±0.50)	20.85 (±0.95)	20.50 (±0.50)	9.55 (±0.55)	68 (±4)
**G12/M9/S9**	16.95 (±0.65)	18.00 (±0.35)	16.75 (±0.80)	9.45 (±0.40)	60 (±1)
**M10/N2.5/S7.5**	15.70 (±1.30)	17.00 (±1.00)	16.30 (±1.00)	9.15 (±0.40)	56 (±3)
**M12.5/S7.5**	15.50 (±0.80)	16.40 (±0.90)	15.05 (±1.00)	8.90 (±0.15)	52 (±2)
**M15/S5**	14.20 (±1.30)	15.05 (±1.30)	13.65 (±2.00)	6.05 (±0.20)	49 (±3)

**Table 5:** RCPT and surface resistivity test results.

Mixture Name	RCPT		Surface Resistivity Test	
	Result (Coulombs)	Coefficient of Variation (%)	Result (kΩ-mm)	Coefficient of Variation (%)
**F10/S10 (Control Mixture)**	165	2.2	386	14.8
**N5/F5/S10**	142	2.6	424	20.8
**N7.5/F2.5/S10**	136	1.4	463	14.4
**N10/S10**	N/A *	N/A *	N/A *	N/A *
**M5/F5/S10**	135	0.9	437	16.8
**M7.5/F2.5/S10**	124	1.5	520	18.9
**M10/S10**	119	2.1	603	8.7
**G2/F8/S10**	162	1.1	395	14.5
**G4/F6/S10**	155	2.0	412	13.1
**M5/N5/S10**	122	1.5	458	15.9
**G2/M6/N2/S10**	121	2.0	431	16.1
**G2/M9/S9**	125	2.4	584	13.8
**G12/M9/S9**	N/A *	N/A *	N/A *	N/A *
**M10/N2.5/S7.5**	162	1.5	435	14.2
**M12.5/S7.5**	160	0.8	440	10.2
**M15/S5**	N/A *	N/A *	N/A *	N/A *

* Not tested since the mixture did not meet UHPC requirements.

## Data Availability

The data presented in this study will be openly available in [Transportation Consortium of South-Central States], project No. 21CNMSU60 entitled “Supplementary Cementitious Materials in UHPC”.

## References

[1] Graybeal B., Tanesi J. (2007). Durability of an Ultrahigh-Performance Concrete. J. Mater. Civ. Eng..

[2] (2017). Standards Practice for Fabricating on Testing Specimens of UHPC.

[3] (2019). Concrete Materials and Methods of Concrete Construction, Annex U-Ultra-High Performance Concrete (UHPC).

[4] (2016). Béton Fibré Ultra-Performant (BFUP)-Matériaux, Dimensionnement et Exécution (Ultra-High Performance Fibre Reinforced Cement-Based Composites [UHPFRC]- Construction Material, Dimensioning and Application).

[5] Graybeal B. (2014). Design and Construction of Field-Cast UHPC Connections.

[6] Akeed M.H., Qaidi S., Ahmed H.U., Faraj R.H., Mohammed A.S., Emad W., Tayeh B.A., Azevedo A.R.G. (2022). Ultra-high performance fiber-reinforced concrete. Part II: Hydration and microstructure. Case Stud. Constr. Mater..

[7] Zhang J., Huang Y., Ma G., Nener B. (2021). Mixture Optimization for Environmental, Economical and Mechanical Objectives in Silica Fume Concrete: A Novel Frame-Work Based on Machine Learning and A New Meta-Heuristic Algorithm. Resour. Conserv. Recycl..

[8] Pedro D., de Brito J., Evangelista L. (2018). Durability Performance of High-Performance Concrete Made with Recycled Aggregates, Fly Ash and Densified Silica Fume. Cem. Concr. Compos..

[9] ACAA (American Coal Ash Association) Fly Ash Use in Concrete Increases Slightly as Overall Coal Ash Recycling Rate Declines. https://acaa-usa.org.

[10] Diaz-Loya I., Juenger M., Seraj S., Minkara R. (2019). Extending Supplementary Cementitious Material Resources: Reclaimed and Remediated Fly Ash and Natural Pozzolans. Cem. Concr. Compos..

[11] Wille K., El-Tawil S., Naaman A.E. (2014). Properties of Strain Hardening Ultra High Performance Fiber Reinforced Concrete (UHP-FRC) Under Direct Tensile Loading. Cem. Concr. Compos..

[12] Wille K., Boisvert-Cotulio C. (2015). Material Efficiency in the Design of Ultra-High Performance Concrete. Constr. Build. Mater..

[13] Karim R., Najimi M., Shafei B. (2019). Assessment of Transport Properties, Volume Stability, and Frost Resistance of Non-Proprietary Ultra-High Performance Concrete. Constr. Build. Mater..

[14] Horák P., Pešková S., Jogl M., Sovják R., Vítek P. (2022). Experimental Investigation of Cohesion between UHPC and NSC Utilising Interface Protrusions. Materials.

[15] Sohail M.G., Kahraman R., Nuaimi N.A., Gencturk B., Alnahhal W. (2021). Durability characteristics of high and ultra-high performance concretes. J. Build. Eng..

[16] Habert G., Denarié E., Šajna A., Rossi P. (2013). Lowering the global warming impact of bridge rehabilitations by using Ultra High Performance Fibre Reinforced Concretes. Cem. Concr. Compos..

[17] American Concrete Institute (ACI) Committee 116 (2000). Cement and Concrete Terminology.

[18] Granata M.F. (2015). Pumice Powder as Filler of Self-Compacting Concrete. Constr. Build. Mater..

[19] Pinarci I., Kocak Y. (2022). Hydration Mechanisms and Mechanical Properties of Pumice Substituted Cementitious Binder. Constr. Build. Mater..

[20] Al-Chaar G.K., Alkadi M., Asteris P.G. (2013). Natural Pozzolan as a Partial Substitute for Cement in Concrete. Open Constr. Build. Technol. J..

[21] Ahmad S., Hakeem I., Maslehuddin M. (2014). Development of UHPC Mixtures Utilizing Natural and Industrial Waste Materials as Partial Replacements of Silica Fume and Sand. Sci. World J..

[22] Ahmad S., Mohaisen K.O., Adekunle S.K., Al-Dulaijan S.U., Maslehuddin M. (2019). Influence of Admixing Natural Pozzolan as Partial Replacement of Cement and Microsilica in UHPC Mixtures. Constr. Build. Mater..

[23] Pezeshkian M., Delnavaz A., Delnavaz M. (2021). Development of UHPC Mixtures Using Natural Zeolite and Glass Sand as Replacements of Silica Fume and Quartz Sand. Eur. J. Environ. Civ. Eng..

[24] Pillay D.L., Olalusi O.B., Kiliswa M.W., Awoyera P.O., Kolawole J.T., Babafemi A.J. (2022). Engineering Performance of Metakaolin Based Concrete. Clean. Eng. Technol..

[25] Tongbo S., Bin W., Lijun Z., Zhifeng C., Scrivener K., Favier A. (2015). Meta-Kaolin for High Performance Concrete. Calcined Clays for Sustainable Concrete.

[26] Rangaraju P.R., Li Z. (2016). Development of UHPC Using Ternary Blends of Ultra-Fine Class F Fly Ash, Meta-kaolin and Portland Cement. Int. Interact. Symp. Ultra-High Perform. Concr..

[27] Tafraoui A., Escadeillas G., Lebaili S., Vidal T. (2009). Metakaolin in the Formulation of UHPC. Constr. Build. Mater..

[28] Tafraoui A., Escadeillas G., Vida T. (2016). Durability of the Ultra High Performances Concrete Containing Metakaolin. Constr. Build. Mater..

[29] Song Q., Yu R., Shui Z., Wang Y., Rao S., Wu S., He Y. (2019). Physical and Chemical Coupling Effect of Metakaolin Induced Chloride Trapping Capacity Variation for Ultra High Performance Fibre Reinforced Concrete (UHPFRC). Constr. Build. Mater..

[30] Gruber K.A., Ramlochan T., Boddy A., Hootan R., Thomas M. (2001). Increasing Concrete Durability with High-Reactivity Metakaolin. Cem. Concr. Compos..

[31] Li Z. (2016). Drying Shrinkage Prediction of Paste Containing Meta-Kaolin and Ultrafine Fly Ash for Developing Ultra-High Performance Concrete. Mater. Today Commun..

[32] Kumar V.S., Ganesan N., Indira P.V. (2021). Engineering Properties of Hybrid Fibre Reinforced Ternary Blend Geopolymer Concrete. J. Compos. Sci..

[33] Kumar V.S., Ganesan N., Indira P.V. (2021). Effect of Hybrid Fibres on the Durability Characteristics of Ternary Blend Geopolymer Concrete. J. Compos. Sci..

[34] Soutsos M.N., Barnett S.J., Bungey J.H., Millard S.G. (2005). Fast Track Construction with High-Strength Concrete Mixes Containing Ground Granulated Blast Furnace Slag. ACI Mater. J..

[35] Tazawa E., Miyazawa S. (1995). Influence of Cement and Admixture on Autogenous Shrinkage of Cement Paste. Cem. Concr. Res..

[36] Ghafari E., Ghahari S.A., Costa H., Júlio E., Portugal A., Durães L. (2016). Effect of Supplementary Cementitious Materials on Autogenous Shrinkage of Ultra-High Performance Concrete. Constr. Build. Mater..

[37] Kim H., Koh t., Pyo S. (2016). Enhancing Flowability and Sustainability of Ultra High Performance Concrete Incorporating High Replacement Levels of Industrial Slags. Constr. Build. Mater..

[38] Lim J.L.G., Raman S.N., Safiuddin M., Zain M.F.M., Hamid R. (2019). Autogenous Shrinkage, Microstructure, and Strength of Ultra-High Performance Concrete Incorporating Carbon Nanofibers. Materials.

[39] Abdulkareem O.M., Fraj A.B., Bouasker M., Khouchaf L., Khelidj A. (2021). Microstructural Investigation of Slag-Blended UHPC: The Effects of Slag Content and Chemical/Thermal Activation. Constr. Build. Mater..

[40] Yalcinkaya C., Copuroglu O. (2021). Hydration Heat, Strength and Microstructure Characteristics of UHPC Containing Blast Furnace Slag. J. Build. Eng..

[41] Yalçınkaya C., Yazıcı H. (2017). Effects of Ambient Temperature and Relative Humidity on Early-Age Shrinkage of UHPC with High-Volume Mineral Admixtures. Constr. Build. Mater..

[42] Toledo W.K., Newtson C.M. Effects of Substrate Texture and Moisture Conditions on Ultra-High Performance Concrete and Silica Fume Concrete Overlay Bond Strengths. Proceedings of the 6th World Multidisciplinary Civil Engineering–Architecture-Urban Planning Symposium (WMCAUS).

[43] Shi C., Wang D., Wu L., Wu Z. (2015). The hydration and microstructure of ultra high-strength concrete with cement–silica fume–slag binder. Cem. Concr. Compos..

[44] Hasan T.M., Gilbert L., Allena S., Owusu-Danquah J., Torres A. (2022). Development of Non-Proprietary Ultra-High Performance Concrete Mixtures. Buildings.

[45] (2020). Standard Test Method for Slump of Hydraulic-Cement Concrete.

[46] (2021). Standard Test Method for Slump Flow of Self-Consolidating Concrete.

[47] (1983). Method for Determination of Compressive Strength of Concrete Cubes. British Standard Testing Concrete Part 116.

[48] (2019). Standard Test Method for Flexural Performance of Fiber-Reinforced Concrete (Using Beam with Third-Point Loading).

[49] (2017). Standard Test Method for Length Change of Hardened Hydraulic-Cement Mortar and Concrete.

[50] (2015). Standard Test Method for Resistance of Concrete to Rapid Freezing and Thawing.

[51] (2019). Standard Test Method for Fundamental Transverse, Longitudinal, and Torsional Resonant Frequencies of Concrete Specimens.

[52] (2022). Standard Test Method for Electrical Indication of Concrete’s Ability to Resist Chloride Ion Penetration.

[53] (2019). Standard Method of Test for Surface Resistivity Indication of Concrete’s Ability to Resist Chloride Ion Penetration.

[54] Mousavinezhad S., Gonzales G.J., Toledo W.K., Garcia J.M., and Newtson C.M. (2022). Mechanical Properties of Ultra-high performance Concrete Containing Natural Pozzolan and Metakaolin. Tran-SET, 2022.

[55] Yazıcı H., Yiğiter H., Karabulut A., Baradan B. (2008). Utilization of Fly Ash and Ground Granulated Blast Furnace Slag as an Alternative Silica Source in Reactive Powder Concrete. Fuel.

[56] Muhd Norhasri M.S., Hamidah M.S., Fadzil A.M., Megawati O. (2016). Inclusion of nano metakaolin as additive in ultra high performance concrete (UHPC). Constr. Build. Mater..

[57] Norhasri M.M., Hamidah M., Fadzil A.M. (2019). Inclusion of nano metaclayed as additive in ultra high performance concrete (UHPC). Constr. Build. Mater..

[58] Newtson C., Weldon B., Garcia J., Mousavinezhad S., Toledo W. (2021). Durability of Concrete Produced with Alternative Supplementary Cementitious Material.

[59] Mo Z., Wang R., Gao X. (2020). Hydration and Mechanical Properties of UHPC Matrix Containing Limestone and Different Levels of Metakaolin. Constr. Build. Mater..

[60] Tanesi J., Graybeal B., Simon M. Effects of Curing Procedure on Freeze-Thaw Durability of Ultra-High Performance Concrete. Proceedings of the 6th International RILEM Symposium on Fibre Reinforced Concretes.

[61] Graybeal B. (2011). Ultra-High Performance Concrete.

[62] Parande A.K., Babu B.R., Karthik M.A., Deepak Kumaar K.K., Palaniswamy N. (2008). Study on Strength and Corrosion Performance for Steel Embedded in Metakaolin Blended Concrete/Mortar. Constr. Build. Mater..

[63] Malakooti A., Thomas R.J., Maguire M. (2019). Investigation of Concrete Electrical Resistivity as a Performance-Based Test.

